# IPNV with high and low virulence: host immune responses and viral mutations during infection

**DOI:** 10.1186/1743-422X-8-396

**Published:** 2011-08-10

**Authors:** Astrid Skjesol, Ingrid Skjæveland, Marianne Elnæs, Gerrit Timmerhaus, Børge N Fredriksen, Sven Martin Jørgensen, Aleksei Krasnov, Jorunn B Jørgensen

**Affiliations:** 1 1 Norwegian College of Fishery Sciences, University of Tromsø N- 9037 Tromsø, Norway; 2 Nofima Marin, P.O. Box 5010, 1432 Ås, Norway

**Keywords:** IPNV, birnavirus, virulence, immune response, fish, non-synonymous changes

## Abstract

**Background:**

Infectious pancreatic necrosis virus (IPNV) is an aquatic member of the *Birnaviridae *family that causes widespread disease in salmonids. IPNV is represented by multiple strains with markedly different virulence. Comparison of isolates reveals hyper variable regions (HVR), which are presumably associated with pathogenicity. However little is known about the rates and modes of sequence divergence and molecular mechanisms that determine virulence. Also how the host response may influence IPNV virulence is poorly described.

**Methods:**

In this study we compared two field isolates of IPNV (NFH-Ar and NFH-El). The sequence changes, replication and mortality were assessed following experimental challenge of Atlantic salmon. Gene expression analyses with qPCR and microarray were applied to examine the immune responses in head kidney.

**Results:**

Significant differences in mortality were observed between the two isolates, and viral load in the pancreas at 13 days post infection (d p.i.) was more than 4 orders of magnitude greater for NFH-Ar in comparison with NFH-El. Sequence comparison of five viral genes from the IPNV isolates revealed different mutation rates and Ka/Ks ratios. A strong tendency towards non-synonymous mutations was found in the HRV of VP2 and in VP3. All mutations in VP5 produced precocious stop codons. Prior to the challenge, NFH-Ar and NFH-El possessed high and low virulence motifs in VP2, respectively. Nucleotide substitutions were noticed already during passage of viruses in CHSE-214 cells and their accumulation continued in the challenged fish. The sequence changes were notably directed towards low virulence. Co-ordinated activation of anti-viral genes with diverse functions (IFN-a1 and c, sensors - Rig-I, MDA-5, TLR8 and 9, signal transducers - Srk2, MyD88, effectors - Mx, galectin 9, galectin binding protein, antigen presentation - b2-microglobulin) was observed at 13 d p.i. (NFH-Ar) and 29 d p.i. (both isolates).

**Conclusions:**

Mortality and expression levels of the immune genes were directly related to the rate of viral replication, which was in turn associated with sequences of viral genes. Rapid changes in the viral genome that dramatically reduced virus proliferation might indicate a higher susceptibility to protective mechanism employed by the host. Disease outbreak and mortality depend on a delicate balance between host defence, regulation of signalling cascades and virus genomic properties.

## Background

Despite vaccination programs, outbreaks of infectious pancreatic necrosis disease (IPN) are frequent in farmed salmon fry and post-smolts. Mortality rates observed in outbreaks vary considerably and have in part been ascribed to the inherited differences in susceptibility of the host [1-3]. Environmental stress [4-7] and the viral strains [8] also influence mortality. Atlantic salmon surviving an IPNV infection may become asymptomatic carriers of the virus for long periods [9,10]. The production of virus may increase under stress, and carriers can shed the virus and infect surrounding fish [11].

The IPN virus (IPNV) is a bi-segmented double-stranded RNA (dsRNA) virus in the family *Birnaviridae *encoding 5 viral proteins. Segment B encodes the RNA-dependent RNA polymerase VP1. Segment A encodes a polyprotein which is cotranslationally cleaved by the viral encoded serine-lysine protease (VP4) releasing the proteins pVP2 and VP3 [12,13]. pVP2 is further processed by host cell proteases to form the mature outer capsid protein VP2 [14], which is the most abundant virus protein and contains the antigenic regions responsible for induction of neutralizing antibodies in the host [15]. VP3 is the inner structural protein, which bound to dsRNA constitutes the ribonucleoprotein core structure [16]. Additionally VP3 is shown to bind VP1 and to self-associate strongly, indicating that it is a matrix protein [17]. An alternative open reading frame (ORF) on Segment A encodes the small, arginine-rich, non-structural protein VP5. The biological function of IPNV VP5 remains to be determined.

The molecular basis of IPNV virulence and its interplay with host antiviral mechanisms are not fully understood. Sano and co-workers [18] were the first to suggest that the virulence of IPNV is associated with Segment A. Several studies using nucleotide sequence analyses have confirmed this and have shown that the VP2 residues 217 and 221 are the major determinant of virulence of IPNV serotype Sp strains. In addition, position 247 was seen as highly variable [19]. Highly virulent isolates possess residues Thr^217 ^and Ala^221^; moderate- to low-virulence strains have Pro^217 ^and Ala^221^, while the strains containing Thr^221 ^are almost avirulent, irrespective of the residue at position 217. IPNV isolates also differ in properties related to replication rate and the ability to cause persistent infections. These characteristics can be attributed to the same amino acids as those determining the virulence [8]. Although some of the factors behind these mechanisms are known there are still many questions to be answered.

During viral infections the initial response of the immune system is the induction of type I interferons (IFN), which mediate antiviral and immunomodulatory activity. In Atlantic salmon three different subtypes of type I IFN have been identified: IFN-a, b and c [20]. IFN-a1 and c are both expressed in head kidney and are induced by poly I:C [20]. IFN-a1 has been shown to provide protection against IPNV in salmonid cells [21,22]. The generation of anti-viral responses during infections requires a rapid viral sensing by pattern recognition receptors (PRRs). Toll-like receptors (TLRs) on the cell-surface or within endosomes recognize single-stranded RNA (ssRNA) and dsRNA [23], while the helicases RIG-I and MDA5 recognize ssRNA and dsRNA in the cytosol [24]. Additionally, dsRNA are recognized by PKR [25]. A number of PRRs have been identified in Atlantic salmon including RIG-I [26], MDA5 (GenBank: EG820831), PKR (GenBank: EF523422), TLR3 [20], TLR8 [27], TLR9 [28] and TLR22 (GenBank: CAJ80696[29] and FM206383). The latter is a dsRNA-specific PRR found exclusively in lower vertebrates [30]. Several studies have shown strong activation of immune genes upon challenge with highly virulent IPNV isolates [31,32], and type I IFNs and the IFN-inducible Mx gene were among the most highly up-regulated genes. However, it remains to find, which PRRs are required for the induction of a systemic type I IFN response during IPNV infections.

Little is known about the relationship between the host responses and the virulence of different IPNV isolates. The latter can be associated with either down-regulation or excessive stimulation of innate immunity. Studies of IPNV infected cell-lines [33,34] have shown inhibition of IFN signaling. In the current work we have assessed immune gene expression changes during an experimental challenge of salmon post-smolts with both a virulent and an avirulent IPNV field strain. In addition to quantitative real-time RT PCR (qPCR) analyses of selected genes we used cDNA microarray, which expanded the repertoire of genes.

The two IPNV isolates used in this study were originally collected from field outbreaks of IPN with 32% and 5% reported mortality, respectively. Initial characterization of the isolates in our lab showed that they both contained the high virulent motif Thr^217 ^Ala^221 ^Thr^247 ^in VP2 defined by Santi et al [19]. A previous bath challenge performed by our group included these isolates, and indicated that they differed substantially in virulence levels reflecting the field mortalities (unpublished). Sequencing of VP2 after this challenge confirmed the Thr^217 ^Ala^221 ^motif in both isolates. However, propagation of the isolates in cell-cultures before the challenge experiment reported here changed the isolate with the initial low mortality to contain a Thr^217 ^Thr^221 ^Thr^247 ^motif (low virulent motif). Santi et al [19] suggested that the Ala-to-Thr substitution at position 221 in VP2 is a molecular determinant for the establishment of a persistent IPNV infection. Thus, we have analyzed the IPNV VP2 nucleotide composition in virus recovered from fish head kidney during the infection. Comparing sequences to the input strains could provide information about the rate and character of virus changes *in vivo*.

## Results

### Two isolates of IPNV gave divergent mortality rates in a bath challenge

**Figure 1:**
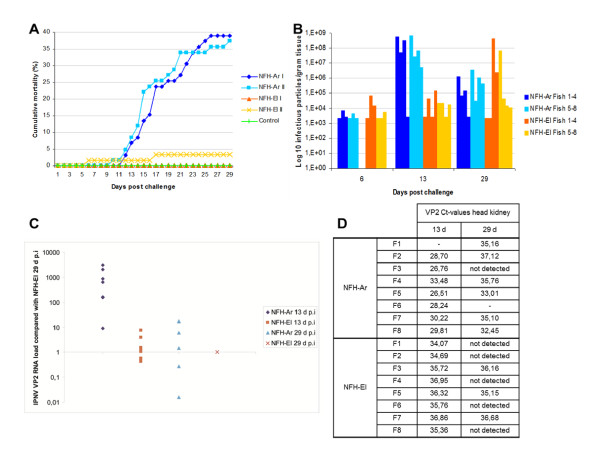
**Cumulative mortality and presence of IPN virus in pancreas and head kidney of Atlantic salmon challenged with two different IPNV strains**. **A**. Percent cumulative mortality is significantly higher in fish infected with NFH-Ar (tank I and tank II indicated with dark and light blue, respectively) than with NFH-El (two tanks indicated with orange or yellow) after bath challenge. **B**. Viral titers in pancreas from 3-4 fish in duplicate tanks at day 6, day 13 and day 29 post challenge, measured by TCID_50_/g tissue. **C**. IPNV VP2 transcript levels relative to levels of NFH-El 29 d.p.i in head kidney measured by qPCR. **D**. IPNVVP2 Ct-values measured in head kidney from individual fish at day 13 and day 29.

Before initiation of this challenge, head kidney from 10 fish was sampled and checked for the presence of IPNV by carrier tests in pools of 3 individuals. The lower detection limit was 0.88 virus particles g^-1 ^tissue. No IPNV positive pools were found. In the bath challenge study, mortality caused by IPNV was initiated 12 days post infection (d p.i.) with the NFH-Ar isolate and there was a rapid increase in mortality until day 21. The experiment was terminated 30 days post IPNV challenge which was one week after the fish in both groups had ceased to die. At that point, fish infected with the NFH-Ar isolate showed far higher cumulative mortality (38.1%, average of two tanks) compared to the NFH-El isolate which gave only 1.6% cumulative mortality on average (Figure 1A). No mortality was observed among the control fish.

### Virus quantification in pancreas and head kidney

The pancreatic tissue was used for quantification of virus as it is one of the primary target organs for IPNV replication. The viral titer was determined as infectious particles per gram tissue from 6 fish infected with each isolate at day 6, and from 8 fish at day 13 and 29. Viral titers increased for both isolates from day 6 to day 13, although for NFH-El (low-mortality groups) the replication was slower compared to NFH-Ar (high-mortality group). A further increase in titer from day 13 to day 29 was observed for NFH-El, whereas NFH-Ar showed decreased titers at 29 d p.i. (Figure 1B). The number of NFH-Ar infectious particles was 3.4 × 10^3^/g in average at day 6, increased to 2.2 × 10^8 ^at day 13 and dropped to 8.5 × 10^5 ^by the third sampling at day 29. Whereas the number of infectious particles for the NFH-El isolate was slightly higher at the first sampling (average 1.6 × 10^4^/g), only increasing to 3.3 × 10^4 ^at day 13 and further leaps to 6.4 × 10^7 ^at day 29. The individual variation was greater in the groups infected with NFH-El than the NFH-Ar infected groups.

The levels of IPNV VP2 RNA in the head kidney of NFH-Ar and NFH-El challenged fish were quantified by qPCR during the time-course. We calculated the relative quantity of VP2 by comparing the expression levels in the different samples to NFH-El sampled at 29 d.p.i. VP2 RNA was detected neither in the control fish nor in the two infected groups at 6 d p.i. The qPCR results for other time-points were overall in agreement with the viral titers determined in pancreas samples from the infected fish. The group infected with NFH-Ar had prevalence of 100% (7/7) at day 13 p.i. with an average 1000 fold higher VP2 RNA load to NFH-El 29 d p.i; while at day 29 p.i. the prevalence was 57% (4/7) and VP2 RNA levels were approximately 7 fold higher compared to NFH-El 29 d p.i. (Figure 1C, D). For NFH-El a decrease in IPNV VP2 RNA levels was observed from 13 d p.i. to 29 d p.i. in head kidney. At day 13 d p.i. the prevalence was 100% (8/8) for NFH-El (2 fold higher virus load than 29 d p.i.), while at day 29 it had decreased to 37.5% (3/8)

### Sequence comparison of the viral genes

**Figure 2:**
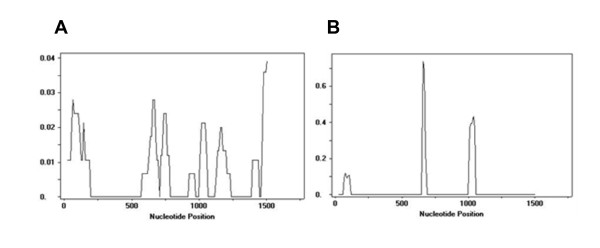
**The highest level of sequence changes in VP2 coincided with the region that is regarded as a determinant of virulence**. **A**. A sliding window plot of mutation frequencies, and **B**. Ka/Ks ratio in VP2 (window length - 50 sites, step size - 10 sites).

**Figure 3:**
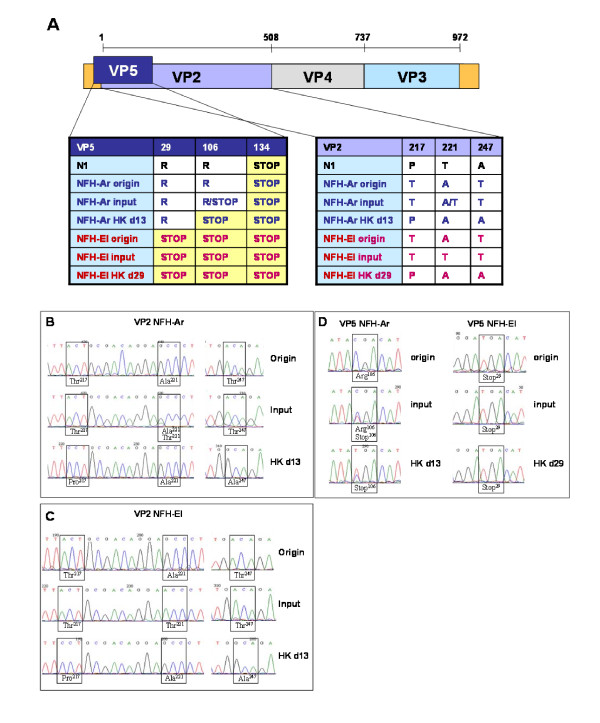
**Sequence analyses of IPNV segment A**. **A**. A schematic presentation of the IPNV genome segment A which encode VP2, VP4, VP3 and VP5. Positions of divergent amino acid are indicated, and the sequences of VP2 and VP5 from the two field isolates NFH-Ar and NFH-El are compared to the non-virulent lab-strain N1 before and after the challenge experiment. **B**. Sequencing chromatograms showing that amino acids in positions 217, 221 and 247 of VP2 were changing rapidly in cell culture and in challenged fish. The PCR-products sequenced from the NFH-Ar strain changed partially from a TAT motif found in the originally infected fish (origin) to a TTT motif after propagation in CHSE-214 cells for the challenge experiment (input). Virus isolated from the fish after the challenge had a PAA motif (HK d13). **C**. The NFH-El strain changed from a TAT found in the original material (origin) to a TTT motif after three passages in CHSE-214 cells (input). Virus isolated from the fish after this challenge (HK d29) had a PAA motif like seen in the NFH-Ar challenged fish. **D**. The ORF of VP5for the NFH-Ar strain changed from a 133 amino acid peptide to a 105 amino acid peptide when a stop codon was introduced in position 106 after the challenge. The NFH-El strain initially had a very short VP5 ORF, only 28 amino acids long, which was maintained after the challenge.

The sequences encoding the individual viral proteins were determined for each of the isolates to evaluate the rate and character of changes and to search for the differences associated with virulence. The virus isolates were sequenced at several stages of the experiment: initially after the field outbreaks, then after propagating the virus in the CHSE-214 cell-line, before the challenge and finally, in the infected fish. The rate of sequence changes was lowest in VP4 (7.4 sites/kb) being 2-2.7 times greater in other genes (Table 1). VP1 showed a higher rate of synonymous (silent) substitutions (Ka/Ks = 0.03). For VP2 and VP3 the non-synonymous (amino-acid replacement) nucleotide substitution rate was much higher (one order of magnitude greater in VP2 and VP3), and its distribution along the sequences strongly suggested presence of hyper variable regions (HVR). Importantly, the first of two HVRs of VP2 with the highest level of Ka/Ks (Figure 2) coincided with the region that is regarded as a determinant of virulence [8,19]. All mutations in VP5 produced premature stop codons. The deduced amino acid sequences for VP2 and VP5 from the two isolates were aligned to each other and to the reference strain N1 before and after the challenge (Figure 3A, B, C and 3D). After sampling from the original field outbreaks (which were reported to give 32% mortality for the NFH-Ar and 5% mortality for the NFH-El isolate) the isolates were passaged once in CHSE-214 cells before sequence analyses. The VP2 sequences revealed a Thr^217^-Ala^221^-Thr^247 ^motif in both isolates (origin) (Figure 3A, B and 3C). Originally, sequencing of VP5 NFH-Ar gave an ORF resulting in a 133 amino acid protein (15.2 kDa predicted), whereas VP5 from NFH-El had a premature stop-codon resulting in a very short ORF, encoding only 28 amino acid residues (3.3 kDa predicted) (Figure 3A and 3D). In the course of this study sequence changes in VP2 and VP5 accumulated continuously, during passage in culture and infection in the fish (Figure 3A). In order to obtain sufficient amount of virus to perform the bath challenge, the isolates were passaged additional times in CHSE-214 cells (2x for NFH-Ar and 3x and NFH-El). Sequencing of this material (challenge input) revealed a change into a Thr^217^-Thr^221^-Thr^247 ^motif in VP2 for the NFH-El and a partial shift from Ala^221 ^to Thr^221 ^in NFH-Ar (Figure 3A, B and 3C). Additionally a partial shift from an Arg (R) in position 106 of VP5 to a stop-codon in this position was observed in NFH-Ar (Figure 3A and 3D). After the challenge viral RNA/cDNA from the head kidney of infected fish (n = 8) was sequenced and the VP2 motifs changed into Pro^217^-Ala^221^-Ala^247 ^in both isolates during the infection process in the fish (Figure 3A, B and 3C). VP5 in NFH-Ar acquired a stop-codon in position 106, resulting in a 12.1 kDa protein, whereas NFH-El maintained it's short (3.3 kDa) VP5 (Figure 3A and 3D).

**Table 1:** Mutation rates in IPNV genome

	VP1	VP2	VP3	VP4	VP5
Length bp	2535	1521	710	679	402
Mutations per 1 kb	14.6	15.8	19.7	7.3	17.4
Non-synonymous mutations	3	12	9	2	7
Synonymous mutations	33	12	5	3	0
Ka/Ks	0.03	0.36	0.40	0.17	

### Gene expression

**Figure 4:**
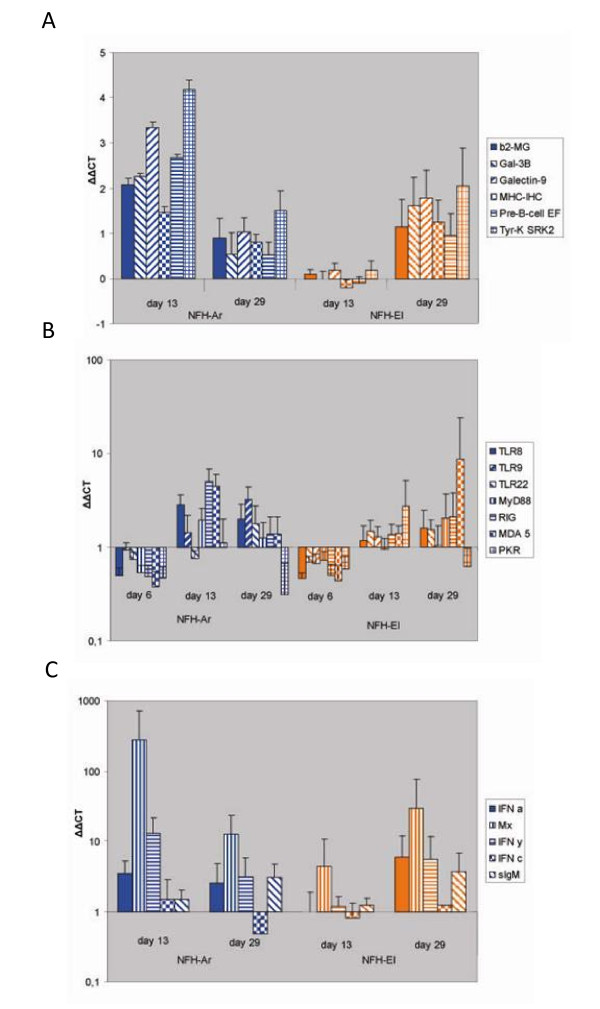
**Regulation of immune pathways in response to infection with the two IPNV isolates analyzed by qPCR**. Data are mean fold change in head kidney transcript levels +/- SE from infected fish relative to controls calculated by the ΔΔCT method, adjusted for PCR efficiency and normalized against the elongation factor. **A**. Validation of microarray results by qPCR on selected immune genes presented in Table 2 (n = 6). **B**. Expression of pattern recognition receptors, MyD88 and PKR analyzed in 8 fish per strain at each time point. **C**. Expression of genes involved in innate immunity/IFN signaling and sIgM analyzed in 8 fish per strain at each time point.

Microarray analyses revealed 211 differentially expressed genes, of which 81 and 130 genes responded to both isolates or to only NFH-Ar respectively; we did not find genes that were regulated exclusively in the NFH-El infected salmon. Both isolates affected genes within different functional groups. Equal down-regulation was observed in genes encoding motor proteins (myosins, tropomyosins, troponins), plasma proteins (plasminogen precursor, coagulation factor X, angiotensin I converting enzyme) and a suite of enzymes involved in xenobiotic metabolism and detoxification (Additional file 1). Similar changes were seen in immune related genes implicated in different processes (Table 2). It is worth noticing that genes involved in biosynthesis of eicosanoids, the inflammatory regulators of lipid origin and several complement factors are down-regulated. At the same time the virus responsive genes (VRG) were up-regulated only in fish infected with NFH-Ar. Some VRGs have well established roles in antiviral responses: antigen presentation (beta-2 microglobulin, MHCI, proteasome subunit), signal transduction downstream of IFN (Jak, STAT) and editing viral RNA (double-stranded RNA-specific adenosine deaminase - ADAR); several more genes are known as IFN-dependent. This group also includes genes with undetermined functions, which however have shown consistent induction in other virus infection studies that used the same microarray [35,36] or the new oligonucleotide platform [37]; these are galectins and galectin binding protein, a tripartite motif protein and SRK2-like tyrosine kinase. Six immune genes were chosen for verification of microarray analyses and results produced with two independent methods were in a good agreement (Figure 4A). At 13 d p.i. significant up-regulation was observed only in salmon challenged with NFH-Ar. At 29 d p.i. expression changes were smaller with no difference between the isolates. The greatest expression changes were seen in SRK2.

**Table 2:** Differentially expressed immune genes between IPNV isolates, as assessed by microarray analyses of head kidney at 13 d p.i.

Accession	Gene	Isolate NFH-Ar	Isolate NFH-El
	**Virus responsive**		

GE828737	Beta-2 microglobulin BA1	*1.46 ± 0.21*	0.31 ± 0.19
CX026208	Beta-2-microglobulin JB1	*1.27 ± 0.24*	0.37 ± 0.12
CA043257	MHC class 1b antigen	*0.91 ± 0.17*	0.16 ± 0.12
CU070775	MHC class I heavy chain-1	*0.98 ± 0.19*	0.21 ± 0.07
BX857730	Proteasome subunit beta type 9	*0.62 ± 0.09*	0.10 ± 0.03
CU071943	Galectin 9-1	*0.60 ± 0.17*	-0.06 ± 0.13
CX035552	Galectin 9-2	*1.50 ± 0.44*	0.03 ± 0.11
CA353586	Galectin 9-3	*2.33 ± 0.41*	0.47 ± 0.10
CA379576	Galectin like 2	*1.14 ± 0.31*	0.19 ± 0.12
CA379898	Galectin like 1	*1.34 ± 0.39*	-0.24 ± 0.09
BX079375	Galectin-3 binding protein	*1.83 ± 0.25*	0.21 ± 0.17
CA367930	Interferon inducible protein 1	0.76 ± 0.17	0.58 ± 0.21
CA363130	Interferon-induced 35 kDa protein	*0.75 ± 0.23*	-0.03 ± 0.06
CA376422	Interferon-induced protein 44-2	*0.70 ± 0.16*	0.17 ± 0.08
CA381440	Double-stranded RNA-specific adenosine deaminase	*0.70 ± 0.16*	0.11 ± 0.09
CA378782	Tyrosine-protein kinase Jak1-2	*0.99 ± 0.31*	-0.28 ± 0.13
CA373850	STAT1	*1.11 ± 0.22*	0.02 ± 0.11
CA376536	Tripartite motif (VRG3)	*1.68 ± 0.19*	0.41 ± 0.12
CA349577	Tyrosine-protein kinase SRK2	*1.85 ± 0.57*	-0.32 ± 0.10

	**Chemokines, cytokines, receptors and transducers**		

CA361535	CC chemokine SCYA106	*1.64 ± 0.40*	0.03 ± 0.12
CA366435	CC chemokine SCYA110-1	-0.91 ± 0.10	-0.77 ± 0.08
CA374135	CCL4	-0.78 ± 0.09	-0.78 ± 0.12
CA378286	Interleukin-1 receptor-associated kinase 1-2	0.71 ± 0.15	0.78 ± 0.13
CA362179	Interleukin-1 receptor-like protein 2	*0.85 ± 0.21*	-0.37 ± 0.12
CA361101	Regulator of G-protein signaling 1-2	0.81 ± 0.18	0.52 ± 0.22
CF752495	Transcription factor jun-B-1	*0.59 ± 0.13*	-0.18 ± 0.10
CA342573	TNF receptor superfamily member 11B	*1.48 ± 0.31*	0.10 ± 0.14

	**Eicosanoid metabolism**		

CA346166	Arachidonate 5-lipoxygenase-1	0.59 ± 0.07	0.78 ± 0.14
BX890112	Cytochrome P450 2F1	-1.03 ± 0.04	-0.94 ± 0.11
EV384586	Cytochrome P450 2K4	-1.08 ± 0.07	-1.06 ± 0.19
CA372428	Leukotriene B4 receptor 1	-0.98 ± 0.10	-1.04 ± 0.12
CA366643	Prostaglandine D synthase	*-1.13 ± 0.20*	0.28 ± 0.21
	**Complement, lectins, effectors**		
CA359451	C type lectin receptor C	-0.72 ± 0.05	-0.65 ± 0.17
CA363676	Complement factor B/C2-B	-0.96 ± 0.08	-0.88 ± 0.11
BX082584	Complement factor H-1	-0.92 ± 0.07	-0.61 ± 0.12
BX075495	High affinity Ig Fc receptor I	0.93 ± 0.29	0.20 ± 0.15
CA367288	Properdin	1.02 ± 0.16	0.89 ± 0.15
CA376803	Serine protease-like protein-2	-0.68 ± 0.15	-0.21 ± 0.15
CR943302	Tolloid-like protein (nephrosin)-1	-0.44 ± 0.11	-1.08 ± 0.30
CA370329	Lysozyme C precursor	*0.71 ± 0.13*	-0.04 ± 0.09
CA385270	Granulins	*0.73 ± 0.14*	0.25 ± 0.02
CF752659	Ferritin heavy chain-1	0.82 ± 0.12	0.56 ± 0.24
CA345780	Lysozyme g-2	-0.90 ± 0.10	-0.49 ± 0.36

	**Apoptosis, cell cycle**		

CX720700	BCL2/adenovirus E1B protein-interacting	1.49 ± 0.29	0.93 ± 0.19
CA366608	CASP8 and FADD-like apoptosis regulator	*1.09 ± 0.11*	0.63 ± 0.14
CA384134	G1/S-specific cyclin D2	*1.98 ± 0.41*	-0.12 ± 0.31
CA377697	Platelet-derived endothelial cell growth factor	0.72 ± 0.15	0.47 ± 0.09
CA376673	Pre-B cell enhancing factor	*1.47 ± 0.34*	0.28 ± 0.11

Data are log_2_-ER ± SE. Significant difference between the study groups (t-test, p < 0.05) is indicated with italics.

The expression of selected immune genes not presented on the microarray was analyzed by qPCR (Figure 4B, C). The TLR8, 9, 22, RIG-I, MDA5 and PKR are implicated in viral detection [30,38] and MyD88 is a TLR adaptor signaling molecule [27]. Interestingly, all these nucleic acid recognition molecules were slightly down-regulated at 6 d p.i. in both infected groups (Figure 4B). At the later time-points, TLR22 did not show difference between the infected fish and controls. PKR was down-regulated in the infected fish both at day 6 and day 29 p.i., while a modest but significant (p < 0.05) increase in PKR levels could be detected at day 13 p.i for the NFH-El isolate. The remaining PRRs tested had expression profiles that were similar to those observed in other VRG (Figure 4A, C). TLR8 was up-regulated in NFH-Ar infected fish at both time-points which was significant (p < 0.05) for 13 d p.i, while expression change of TLR9 was significant (p < 0.05) at 29 d p.i. The cytoplasmic RNA sensors RIG-I and MDA5 showed 5-fold induction at 13 d p.i. in the NFH-Ar group which was significant (p < 0.05) for RIG-I. MDA5 also showed a large increase at 29 d p.i. in two individuals infected with NFH-El.

**Figure 5:**
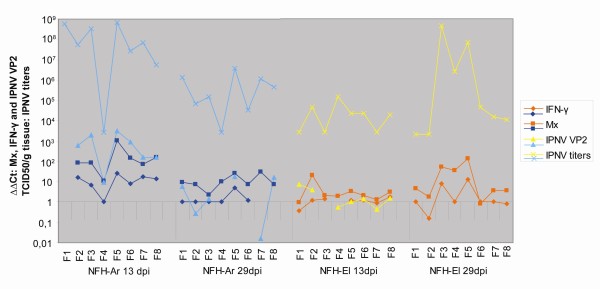
**Variations in immune gene expression and virus levels between individuals**. Transcript levels of Mx, IFN-γ and IPNV VP2 in head kidney and titers of IPNV in pancreas are shown at individual levels for the two IPNV isolates. The Mx and IFN-γ transcript levels are compared to uninfected control fish, while IPNV VP2 transcripts are compared to the NFH-El group 29d.p.i, IPNV titers were calculated by end point titration and results show TCID_50_/g tissue. For some individuals the transcript levels were undetected and are thus not presented.

The antiviral genes Mx and IFN-γ (type II IFN) were the most highly induced transcripts in the study, and the NFH-Ar infected fish showed the greatest expression changes at 13 d p.i.; 280-fold for Mx and a significant (p < 0.05) 13-fold for IFN-γ (Figure 4C). Their expression levels decreased at 29 d p.i. to 13- and 3-fold up-regulation respectively (Figure 4C). In the NFH-El group Mx and IFN-γ showed highest expression at day 29 p.i. with 29- and 5-fold up-regulation respectively. As illustrated in Figure 5, exemplified by Mx and IFN-γ, there were variations in immune gene expression between individuals in both groups. Nevertheless, for most individuals the immune gene expression mirrored the virus levels, both the IPNV titers in pancreas and VP2 transcript levels in head kidney. Three type I IFN genes had different expression profiles during the challenge. While IFN-c did not respond to infection, IFN-a1 showed a moderate up-regulation both at day 13 (3.5-fold) and day 29 p.i. (2.5-fold) in NFH-Ar group, and at day 29 p.i. in the NFH-El group (5.9 fold). Expression of IFN-b in the head kidney was consistently low or undetected; however, an induction was observed at 13 d p.i. in three of eight NFH-Ar-infected fish (results not shown). Secreted IgM was slightly up-regulated at 29 d p.i for both isolates at similar levels (about 3-fold).

## Discussion

IPNV isolates have been reported to vary considerably in their virulence and pathogenicity for Atlantic salmon [18,19]. Previous studies have indicated that a combination of IPNV virulence and host pathogen interactions determines the outcome of IPNV infections. Occurrence of multiple strains indicates rapid changes of the virus but little is known about the rate and character of mutations. It also remains undefined if susceptibility of salmon to IPNV could be associated with low or excessive immune responses. In this study two Norwegian field isolates of IPNV with marked difference in mortality were applied for experimental infection of Atlantic salmon. For the first time the sequence changes and immune responses to low and high virulence strain were compared within a single challenge test.

Previous studies have identified the outer capsid protein VP2 as the main determinant of IPNV virulence as it comprises all the neutralizing epitopes and cell attachment sites that determine host or cell specificity [15]. The amino acid signature associated with virulence of different strains is also identified within the VP2 region [8,19,39]. The sequences of the two isolates in this study were determined before and after the challenge. Initially NFH-Ar had Thr^217^-Ala^221^-Thr^247 ^while NFH-El had Thr^217^-Thr^221^-Thr^247^, implying high and low virulence of NFH-Ar and NFH-El respectively (Figure 3A). Thus the results from this study are in accordance with previously reports of the virulence motifs within VP2 [19]. Rapid changes were seen during passage in cell culture and also during infection in the fish. After the challenge both isolates acquired Pro^217^-Ala^221^-Ala^247 ^motif in VP2 associated with the moderate to low virulence [19]. Sequence analyses revealed a remarkably high rate of non-synonymous substitutions in the HVR containing the virulence motif. Commonly, high Ka/Ks ratios point to the divergent selection meaning that sequence changes increase the rate of reproduction. However in this study we observed accumulation of mutations that correlated with a reduction in virus proliferation, thus being favorable for the fish. End of mortality in NFH-Ar group coincided with the loss of the high virulence motif. To explain this finding, virus modification as the host's defense mechanisms can be hypothesized. Virus editing is a rapidly expanding research area. At present, the best studied actors are adenosine deaminases (ADARs) that target regions of dsRNA, converting adenosine (A) to inosine (I) resulting in an A to guanosine (G) change after second strand synthesis [40]. ADARs target mRNAs, transposable elements and RNA viruses' genomes. In mammals, several families of viruses show A to G mutations thought to be caused by ADARs [41]. The induction of ADAR in the NFH-Ar infected fish observed in the microarray in this study might indicate a possible role for ADAR in editing IPNV. Most of the changes observed in our study, disregarding whether synonymous or non-synonymous, were from A to G or from T to C (or *vice versa*) associated with deamination (results not shown). However, whether the mutations in IPNV detected in this study are caused by salmon ADARs is an interesting question that needs to be addressed in future studies.

The two virus isolates used in this study showed greater discrepancies in the VP5 region than previously described isolates. The change of VP5 into a shorter protein in the NFH-Ar infected fish might imply a more benign virus since the longer 15.2 kDa VP5 protein was shown to have a potential antagonistic effect on the IFN response by inhibiting IFN-induced expression from the Mx promoter [34] and might thereby benefit the viral replication. However, the domains responsible for this function have not been mapped and can still be present in the shorter 12.1 kDa version. An early stop-codon located in NFH-El leaves this isolate with a severely truncated form of VP5, only 28 amino acids long, and to our knowledge such a mutation has not been reported in other surveys of IPNV field isolates. It has been suggested that VP5 has an anti-apoptotic function, which is probably not essential for the virulence or persistence of the virus [42,43]. Functional significance of the observed changes in VP5 remained unclear. The amino acid sequences of VP1 ORF were identical between the two isolates throughout the study and VP4 were subject to very few mutations.

The mortality rates associated with the structural differences described above may be linked to the ability of virus to invade and replicate within the host cells and/or the scale and character of immune responses. Association between mortality and the virus titers was obvious (Figure 1). NFH-El characterized with low replication was avirulent while NFH-Ar infection was fatal at high rate of proliferation at 13 d p.i. To assess the immune responses, we used expression profiling with microarray and qPCR analyses of genes with well-established roles (IFNs, Mx and PRRs) and both approaches produced similar results. The expression levels of VRGs were apparently mirroring the viral titers and expression levels of the IPNV VP2. At 13 d p.i. VRG were induced in salmon infected with NFH-Ar but not with NFH-El consistently with the difference of virus titers. Up-regulation of VRG in NFH-E1 infected fish at 29 d p.i. was in line with the slight increase of virus titer and did not affect mortality. It is likely that the slower replication and concomitant slower spread of the NFH-El strain may allow time for a systemic induction of the host anti-viral system, including adaptive responses.

The IFN system is believed to have a crucial role in the first line of defense against virus infections, and *in vitro *studies have demonstrated that IPNV replication in cell cultures is efficiently inhibited by salmon IFN-a1 [21,22]. Additionally, injection of synthetic IFN-inducers like CpG and poly I:C induce protection against IPNV in Atlantic salmon [44]. In this study IFN-a1 was induced by both viral strains and major up-regulation was seen in the IFN-a1 dependent gene Mx. IFN-c, was not induced and even slightly down-regulated at 29 d p.i. for NFH-Ar. IFN-c is suggested to have a separate regulation from a and b and can be produced by a different cell population than IFN-a1 [20]. IFN-b was not detected in this study or showed consistently low expression levels (data not shown). Despite the suggested role of type I IFN in restraining virus production, results in our lab has demonstrated that IFN-a1 does not completely inhibit IPNV growth but causes a delay in viral protein synthesis [34]. Furthermore, our data suggest that IPNV-encoded proteins may be involved in weakening of IFN signaling [34]. As a result high levels of viral proteins may impair the activity of IFN-induced genes, thus the higher replication rate of NFH-Ar compared to NFH-El may cause a more potent IFN-antagonizing effect of the NFH-Ar strain. However when interpreting the results from live pathogen challenges, it is important to keep in mind the complexity of such studies, where it is not straight forward whether an observed response is a strategy employed by the virus for its own benefit or a response by the host to control the virus.

Although innate immunity by itself represents a powerful system to combat viral invaders, many infections can only be cleared in combination with adaptive immunity. In this regard type I IFNs are known to promote the adaptive arm including both T cell mediated cellular responses and antibody production [45,46]. It is likely that the reduction of virus titers by 29 d p.i. could be associated with the onset of adaptive immune responses. Unlike the genes implicated in the innate immunity, sIgM showed no expression changes at 13 d p.i. and was slightly induced at 29 d p.i.

Results of this study added knowledge to the understanding of the immune responses after IPNV infections. Expression of a panel of PRRs was assessed including several recently identified genes. TLR8, 9 RIG-I and MDA5 showed up-regulation and followed the same trend as other immune genes. Earlier we observed a modest increase of TLR8 and 9 expression during stimulation and infection [27,28]. TLR22 is reported to recognize dsRNA in pufferfish and when over-expressed it induces type I IFN expression upon IPNV infection, which suggests a possible role for TLR22 in protection against IPNV [30]. However, TLR22 was not induced by IPNV in this study. Unexpectedly, PKR, an IFN-inducible gene, was down-regulated at all time points except 13 d p.i. (Figure 4A). This was in contrast to Mx, another IFN-inducible gene and IFN-a1, which were up-regulated. Functional studies of salmon PKR have to our knowledge not been reported, however flounder PKR was up-regulated both *in vitro *and *in vivo *by a negative single stranded RNA virus (SMRV), which also induced Mx expression *in vitro *[47]. PRRs, PKR and MyD88 were down-regulated at 6 d p.i. in both study groups. This could be explained by migration of leukocytes expressing the PRRs from the head-kidney into the bloodstream at early time-points of the infection.

IFN-γ was together with Mx the most highly induced immune gene in this study, and high levels of IFN-γ has been reported in other IPNV challenge experiments [31]. IFN-γ is regarded as a typical Th1 cytokine which bridges the innate and adaptive immune responses. Fish IFN-γ share several functional properties with mammalian IFN-γ including macrophage activation [48-50] and rainbow trout IFN-γ is shown to signal through STAT1 [50,51]. Recently, we have observed antiviral activity against IPNV by IFN-γ, although the effect was not as pronounced as described for IFN-a1 [52]. Like in mammals, fish IFN-γ plays diverse roles in different facets of the immune system, and the increased levels of IFN-γ upon IPNV challenge detected here suggest anti-IPNV activity.

## Conclusions

Our study aimed at investigation of different mortality associated with two IPNV isolates. High incidence of death of NFH-Ar infected salmon was in line with the initial presence of the virulence motif in VP2 and higher virus titers in the head kidney and pancreas compared to NHF-El. PPRs, IFNs and ISGs were induced to a higher magnitude by the high virulent strain NFH-Ar and correlated with the viral load in the head kidney. Subsequent decrease of virus titers and mortality could be due to the sequence changes in the hyper variable regions of IPNV together with the development of acquired immunity in fish.

## Methods

### Virus isolates

Two IPNV isolates, referred to as NFH-Ar and NFH-El, collected from field outbreaks of IPN in 2004 were used in this study. Veterinarians had diagnosed IPN based on clinical observations and by use of an agglutination assay (Phadebact coating kit, Boule Diagnostics AB). The NFH-Ar isolate, obtained from an IPN outbreak in Frøya, Norway in 2004, was reported to give 32% mortality, while the NFH-El, obtained from an IPN outbreak in Alta, Norway in 2004, was reported to give 5% mortality. Head kidney samples collected during these outbreaks were sent to our lab and tissue homogenates were made and inoculated on CHSE-214 cells for propagation and sequencing. For NFH-Ar a second, and for NFH-El a third cell-culture passage of the virus strain was used in the present experiment. The input strains were sequenced before challenge.

### IPNV challenge of Atlantic salmon

The challenge was carried out at Tromsø Aquaculture Research Station (Tromsø, Norway). Non-vaccinated Atlantic salmon, *Salmo salar *L., strain Aquagen IPNV sensitive (Aquagen, Kyrksæterøra, Norway), with an average size of 51 g, was used for the challenge two days after transfer to seawater. Bath challenge was performed as described by Johansen and Sommer [53] using an infectious dose of TCID_50_/ml = 5 × 10^5 ^of the field isolates NFH-Ar and NFH-El. Two parallel tanks supplied with 200 L of 10°C seawater were used for each isolate. Each tank contained 70 fish and a separate tank was used for 70 uninfected control fish. The fish were fed daily on commercial feed. The experiment was terminated 30 days after challenge.

### Sampling

Mortality was monitored throughout the experiment by counting dead fish. An IPNV rapid agglutination kit (Phadebact coating kit, Boule Diagnostics AB) was used on head kidney samples from dead fish to verify IPN as the cause of death. Percent cumulative mortality in infected fish was calculated compared to control fish using GraphPad Prism 4.0 tool for statistical analyses. Sampling was performed on surviving fish. Pancreatic tissue and head kidney were aseptically removed from 4 fish in each tank at 6, 13 and 29 d p.i. Pancreatic tissue was stored in L-15 medium on ice until frozen at -80°C, whereas head kidney samples were stored in RNAlater^® ^(Ambion) at -80°C.

### Quantification of IPNV by titration

Pancreatic tissue was homogenized using an Ultra Thurrax T25 basic crusher (IKA-WERKE). Homogenized tissue was diluted to 5% in Eagle's minimum essential medium (EMEM) (Gibco) supplemented with 100 μg/ml streptomycin, 60 μg/ml penicillin, 2 mM L-glutamine and 1% non-essential amino acids, before centrifuged at 15000 × g for 5 min at 4°C. Homogenates were inoculated onto CHSE-214 cells in 96 wells plates at a starting concentration of 0.5% (w/v). Individual viral titers were determined by end-point titration using 10-fold dilutions and 2 replicates and calculated by the TCID_50 _method [54].

### RNA isolation and cDNA synthesis

For analyses of cellular and viral gene expression total RNA from head kidney sampled from 8 challenged and 4 control fish at each time point, was isolated using a combination of Trizol and PureLink RNA Kit (Invitrogen). RNA quality was assessed on an agarose gel and the quantity determined by NanoDrop 2000 spectrophotometry (Thermo Scientific). No RNA degradation was observed. After isolation, the RNA was DNase treated applying TURBO DNase (Ambion). cDNA was synthesized in a 25 μl reaction from 200 ng DNase treated total RNA primed with random hexamers (TaqMan Reverse Transcription Reagents kit, Applied Biosystems). The manufacturer's protocol was followed. For isolation of viral RNA after passage in cell culture QIAamp Viral RNA Mini Kit (Qiagen) was used according to the manufacturer's instructions.

### Sequencing of virus isolates

Each virus isolate was sequenced from virus derived material under the following conditions; origin (virus homogenates from head kidney pooled from 2 fish in field outbreak passaged 1× in cell culture), input (virus homogenates from head kidney pooled from 2 fish in field outbreak passaged 2-3× in cell culture), during infection(head kidney from day 13 (NFH-Ar) or day 29 (NFH-El) infected fish, 8 pooled individuals from each group). Primers specific for each of the genes encoding the five IPNV proteins were used to amplify the individual genes (from head kidney cDNA pooled from 8 fish or cDNA obtained from head kidney virus homogenates from 2 fish passaged in cell culture) in a PCR reaction using Pfu Turbo Hotstart DNA polymerase (Stratagene). The PCR-products were sequenced in both directions using the same primers and BigDye3.1 chemistry and a 3100 gene analyzer. The sequences were aligned with BioEdit 7.0.5 [55] and DnaSP V5 [56] was used for evaluation of mutation rates and search for HVR.

### Microarray analyses

Microarray analyses were performed at 13 d p.i. on head kidney samples of salmon infected with isolates NFH-Ar and NFH-El (5 individuals from each group, one microarray per individual). The salmonid fish microarray (SFA 2.0 or immunochip, Geo Omnibus GPL6154) contains 1800 unique clones printed each in 6 spot replicates. Pooled samples of uninfected salmon (equal amounts of RNA, n = 4) were used as a common reference. Test and reference RNA (10 μg) were labeled with the fluorescent dyes Cy5-dUTP and Cy3-dUTP respectively (Amersham Pharmacia), which were incorporated in cDNA using the SuperScript™ Indirect cDNA Labeling System (Invitrogen). Synthesis of cDNA was performed at 46°C for 3 hours in a 23 μl reaction volume, following RNA degradation with 2.5 M NaOH at 37°C for 15 min and alkaline neutralization with 2 M Hepes. Labeled cDNA was combined and purified with Microcon YM30 (Millipore). Microarray slides were pre-treated with 1% BSA fraction V, 5× SSC and 0.1% SDS for 30 min at 50°C and then washed with 2× SSC (3 min) followed by 0.2× SSC (3 min) at room temperature and hybridized over-night at 60°C in a cocktail containing 1.3× Denhardt's, 3× SSC, 0.3% SDS, 0.67 μg/μl polyadenylate and 1.4 μg/μl yeast tRNA. After hybridization slides were washed at room temperature in 0.5 × SSC and 0.1% SDS (15 min), 0.5 × SSC and 0.01% SDS (15 min), 0.06 × SSC (2 min) and 0.06 × SSC (1 min). Scanning was performed with GenePix 4100A and images were processed with GenePix 6.0 (Molecular Devices). The spots were filtered by criterion (*I*-*B*)/(S*_I_*+S*_B_*) ≥ 0.6, where *I *and *B *are the mean signal and background intensities and S*_I_*, S*_B _*are the standard deviations. Low quality spots were excluded from analyses and genes with less than 3 high quality spots on a slide were discarded. After subtraction of median background from median signal intensities, the expression ratios (ER) were calculated. Lowess normalization was performed first for the whole slide and next for twelve rows and four columns per slide. Statistical analyses were performed in two stages. First, technical accuracy was assessed by difference of log_2_-ER from zero in six spot replicates and genes with significant changes (Student's t-test, p < 0.05) in at least 3 of 5 individuals per group were selected. Next, analysis of biological replicates was carried out and differential expression was assessed by the mean fold change (> 1.5) and difference from control (one sample t-test, p < 0.05).

### Quantitative real-time RT PCR (qPCR) analyses

PCR primers used with Sybr Green assay were designed using Vector NTI (Invitrogen) and synthesized by Invitrogen (table 3). The amplicon lengths set to be between 50 and 200 bases were checked on 1.5% agarose gel. TaqMan assays employing a hydrolysis probe were designed using Assays-by-design (Applied Biosystems) and synthesized by Applied Biosystems. PCR efficiency was calculated from tenfold serial dilutions of cDNA for each assay in triplicates. qPCR assays were conducted using 2× SYBR^® ^Green Master Mix (Roche Diagnostics) in an optimized 12 μl reaction volume, using 1:10 diluted cDNA, with primer concentrations of 0.4-0.6 μM. PCR was performed in duplicate in 96-well optical plates on Light Cycler 480 (Roche Diagnostics) under the following conditions: 95°C for 5 min (pre-incubation), 95°C for 5 sec, 60°C for 15 sec, 72°C for 15 sec (amplification), 95°C for 5 sec, and 65°C for 1 min (melting curve). Forty cycles were performed. Assays employing a hydrolysis probe was conducted in a 20 μl reaction using 2.5 μl 1:10 fold diluted cDNA as template and 2× TaqMan Fast Universal PCR Master mix (Applied Biosystems). The expression of mRNA was measured in an ABI Prism 7500 FAST Cycler (Applied Biosystems) and the amplification profile was: 95°C for 20 sec followed by 40 cycles of 95°C for 3 sec and 60°C for 30 sec. PCR analyses of the SybrGreen assays for RIG-I, MDA5 and PKR at 13 and 29 d p.i. was also performed at the ABI Prism 7500 FAST Cycler. Relative expression of mRNA was evaluated by ΔΔCT, adjusted for PCR efficiency and normalized against the elongation factor (EF1AB). IPNV VP2 was undetected in control samples and at 6 d p.i., in order to calculate the ΔΔCT values the average of the NFH-El infected fish sampled at 29 d p.i was used as calibrator. Results were analyzed with ANOVA followed with Newman - Keuls test (p < 0.05). (See Table 1 for primers and probes used in the qPCR assays).

**Table 3:** Primers and probes used for qPCR analyses

Genes	Assay	Primer	Sequence (5'-3')	PCR efficiency	GenBank accession #
**EF1aB**	TaqMan	Forward Reverse Probe	TGCCCCTCCAGGATGTCTAC CACGGCCCACAGGTACTG AAATCGGCGGTATTGG	2.0	BG933897
**IFN-c**	TaqMan	Forward Reverse Probe	TGGGCAGTGTGGATACAAGTG CTGCAATGTTCCCAAAGTACGTATT CTGTCCTGATGAGATAAT	2.0	EU735545 EU735547-50
**IFN-a1**	TaqMan	Forward Reverse Probe	CCTTTCCCTGCTGGACCA TGTCTGTAAAGGGATGTTGGGAAAA CTTTGTGATATCTCCTCCCATC	1.94	AY2169594 AY2169595
**IFN-γ**	TaqMan	Forward Reverse Probe	AAGGGCTGTGATGTGTTTCTG TGTACTGAGCGGCATTACTCC TTGATGGGCTGGATGACTTTAGGA	2.0	AY795563
**Mx1/2 e/e**	TaqMan	Forward Reverse Probe	GATGCTGCACCTCAAGTCCTATTA CGGATCACCATGGGAATCTGA CAGGATATCCAGTCAACGTT	2.0	U66475 U66476
**MyD88**	TaqMan	Forward Reverse Probe	GACAAAGTTTGCCCTCAGTCTCT CCGTCAGGAACCTCAGGATACT CTGGTGCCCGGAGCAA	1.84	EF672332
**TLR8**	TaqMan	Forward Reverse Probe	ACCAAAACCACTAATGACATCATCTTCA TGGTGATGCCATCAGGTATGTTT CTCAGTCGACGCTCCTC	1.7	FJ467615
**TLR9**	TaqMan	Forward Reverse Probe	TCTATGGCTGGGATGTCTGGTA CAGTTGTGAGTAGCCCTTGTGT CAGCACCTGGAAGCAG	1.81	EF672331
**TLR22**	TaqMan	Forward Reverse Probe	ATTTATCCCGGAATCCATGTATCACG CCACAGTAGGCGATGTCTAACA CCTCAAGATAAGGAAGAACAT	1.98	AM233509
**VP2 (IPNV)**	TaqMan	Forward Reverse Probe	GCCAAGATGACCCAGTCCAT TGACAGCTTGACCCTGGTGAT CCGACCGAGAACAT	2.0	AJ877117
**Beta 2 microglobulin**	SybrGreen	Forward Reverse	TCGTTGTACTTGTGCTCATTTACAGC CAGGGTATTCTTATCTCCAAAGTTGC	1,6	BX076608
**Galectin-3 binding protein**	SybrGreen	Forward Reverse	CCAGACCAACAGTGTTCACTTCAGC ACGTGAAAGACATACCTGCCCTCAC	1,84	BX079375
**Galectin-9**	SybrGreen	Forward Reverse	GTCCTGTCTATTGCCTTCTCCAACC GGTTTCGTTGACCACTGTGTGGA	1,76	CU071943
**MHC-IHC**	SybrGreen	Forward Reverse	CTGCATTGAGTGGCTGAAGA GGTGATCTTGTCCGTCTTTC	1,72	CU070775
**Pre-B cell ef**	SybrGreen	Forward Reverse	GACTTCAATTTCCTGCTGGCTA CTGCTTGTAATGTGTGACCT	1.77	CA376673
**RIG-I**	SybrGreen	Forward Reverse	GACGGTCAGCAGGGTGTACT CCCGTGTCCTAACGAACAGT	1.97	DY714827
**MDA5**	SybrGreen	Forward Reverse	CAGAGGTGGGGTTCAATGAT AGCTCGCTCCACTTGTTGAT	1.92	FN396357
**PKR**	SybrGreen	Forward Reverse	TTCCTGCATGGACTTGACTG GTGAGGAACCGGTGTTCTGT	1.87	BT046111
**SRK2**	SybrGreen	Forward Reverse	TAGACATGGCACCATGGACCCTC GGGTTCTTCAGTGCAGACAGCCA	1.81	CA349577

## Competing interests

The authors declare that they have no competing interests.

## Authors' contributions

AS performed most of the sequencing, assisted with the challenge experiment and quantification of virus, in addition he drafted the manuscript together with IS. qPCR analyses were performed by IS and GT. ME and BNF performed the challenge experiment and some of the sequencing and virus quantification. The microarray analyzes were done by SMJ and GT. AK contributed to this work with gene expression and sequence data and drafting the manuscript. JBJ designed the experiments, analyzed data and contributed to finalizing the manuscript. All authors read and approved the final manuscript.

## Supplementary Material

Additional file 1 **Differentially expressed genes**. The genes were selected as described in Methods. The data provided represent the log2 (Expression ratios) in IPNV infected salmon and control. Click here for file
